# Clinical relevance of breast and gastric cancer-associated polymorphisms as potential susceptibility markers for oral clefts in the Brazilian population

**DOI:** 10.1186/s12881-017-0390-y

**Published:** 2017-04-04

**Authors:** Renato Assis Machado, Edimilson Martins de Freitas, Sibele Nascimento de Aquino, Daniella Reis B. Martelli, Mário Sérgio Oliveira Swerts, Silvia Regina de Almeida Reis, Darlene Camati Persuhn, Helenara Salvati Bertolossi Moreira, Verônica Oliveira Dias, Ricardo D. Coletta, Hercílio Martelli-Júnior

**Affiliations:** 1 Department of Oral Diagnosis, Dental School, State University of Campinas, Piracicaba, São Paulo Brazil; 2 Stomatology Clinic, Dental School, State University of Montes Claros, Montes Claros, Minas Gerais Brazil; 3 School of Dentistry, Federal University of Juiz de Fora, Governador Valadares, Minas Gerais Brazil; 4 Center for Rehabilitation of Craniofacial Anomalies, University of José Rosário Vellano, Alfenas, Minas Gerais Brazil; 5 Department of Basic Science, Bahiana School of Medicine and Public Health, Salvador, Bahia Brazil; 6 Molecular Biology Departament, Federal University of Paraíba, João Pessoa, Paraíba Brazil; 7 Department of Physiotherapy, State University of Western Paraná, Cascavel, Paraná Brazil

**Keywords:** Nonsyndromic cleft lip with or without cleft palate, Breast cancer, Gastric cancer, *AXIN2*, *CDH1*

## Abstract

**Background:**

Epidemiological studies have indicated a higher incidence of breast and gastric cancer in patients with nonsyndromic cleft lip with or without cleft palate (NSCL ± P) and their relatives, which can be based on similar genetic triggers segregated within family with NSCL ± P.

**Methods:**

This multicenter study evaluated the association of 9 single nucleotide polymorphisms (SNP) in *AXIN2* and *CDH1*, representing genes consistently altered in breast and gastric tumors, with NSCL ± P in 223 trios (father, mother and patient with NSCL ± P) by transmission disequilibrium test (TDT).

**Results:**

Our results showed that the minor A allele of rs7210356 (*p* = 0.01) and the T-G-G-A-G haplotype formed by rs7591, rs7210356, rs4791171, rs11079571 and rs3923087 SNPs (*p* = 0.03) in *AXIN2* were significantly under-transmitted to patients with NSCL ± P. In *CDH1* gene, the C-G-A-A and A-G-A-G haplotypes composed by rs16260, rs9929218, rs7186053 and rs4783573 polymorphisms were respectively over-transmitted (*p* = 0.01) and under-transmitted (*p* = 0.008) from parents to the children with NSCL ± P.

**Conclusions:**

The results suggest that polymorphic variants in *AXIN2* and *CDH1* may be associated with NSCL ± P susceptibility, and reinforce the putative link between cancer and oral clefts.

**Electronic supplementary material:**

The online version of this article (doi:10.1186/s12881-017-0390-y) contains supplementary material, which is available to authorized users.

## Background

Oral clefts are the most common congenital craniofacial abnormalities in humans [1, 2]. About 70% of cases occur as a nonsyndromic form, and the remaining 30% are associated with Mendelian disorders or chromosomal, teratogenic and sporadic conditions [3, 4]. Nonsyndromic oral clefts are traditionally divided in cleft lip only (NSCLO), cleft lip and palate (NSCLP) and cleft palate only (NSCPO), however, as there are similarities in both epidemiologic features and embryologic timing for both NSCLO and NSCLP, they are considered variants of the same defect and grouped together to form the group cleft lip with or without cleft palate (NSCL ± P) [5, 6]. Oral clefts affect approximately 1 in 500–2000 live births, with variable prevalence around the world, often attributed to ethnic and environmental differences. American and Asian populations have a high prevalence (1:500), populations from Europe have intermediate prevalence (1:1000) and the lowest prevalence is observed in Africans and their descendants (1:2500) [7]. Previous studies have revealed that NSCL ± P etiology is dependent of interactions between environmental and genetic risk factors [8, 9]. In fact, epidemiological studies and analysis of animal models have provided evidences of a putative participation of agrotoxics, cigarette smoking, consumption of alcohol and drugs, malnutrition and some viral infections in etiology of NSCL ± P [10, 11]. Genetic linkage and genome-wide association studies (GWAS) have described several genes and loci in association with NSCL ± P, including *IRF6*, *VAX1*, *MSX1*, *FOXE1*, *MYH9*, *MAFB*, *ABCA4*, *BMP4*, *FGFR2*, *TGFA*, *TGFB3*, *MTHFR*, *GSTT1*, *PDGFC*, *FGF8*, *PVRL1*, *SUMO1*, *CRISPLD2* and the regions 8q24 and 17q22 [1, 12, 13].

Epidemiological studies have shown a high risk of oral clefts in families with cancer, while others have provided consistent data about increased risk of cancer in patients with oral cleft and their relatives [14–18]. This association has been explicated because specific cancers, including those of breast and stomach, and NSCL ± P may share similar genetic etiology [14, 19–21]. The Wingless/integrase-1 (WNT) signaling pathway is an evolutionarily conserved pathway with major roles in cell migration, cell polarity, cell fate determination and organogenesis during embryonic development, including orofacial morphogenesis [22–24], and aberrant expression of several WNT family members is common in cancers [25, 26]. The canonical WNT signaling is mediated by the translocation of β-catenin to the nucleus, where it activates transcription of several factors regulating proliferation and differentiation, while noncanonical WNT signaling is β-catenin independent [27].

The axis inhibition protein 2 (*AXIN2*), a member of the WNT pathway, is associated with the development and progression of breast cancers [21, 28], and polymorphisms in *AXIN2* are associated with increased risk for breast cancer in premenopausal women [29]. E-cadherin, encoded by the *CDH1* (*cadherin-1*) gene, is a transmembrane glycoprotein playing a crucial role in maintaining cell-to-cell adhesion [30]. E-cadherin has been reported to be a tumor suppressor and to be downregulated in gastric cancer [31–33]. Germline *CDH1* mutations confer an increased risk of developing both gastric and breast cancer [34]. Genetics studies revealed that polymorphic variants in *AXIN2* and *CDH1* are risk factors for NSCL ± P and tooth agenesis [35–40]. In this study, we postulated that single nucleotide polymorphisms (SNP) in genes associated with breast and gastric pathogenesis (*AXIN2* and *CDH1*) might be related to the risk of NSCL ± P. Thus, we evaluated the association of 9 SNPs in *CDH1* and *AXIN2* with the risk of NSCL ± P using a family-based study design (case-parent trios). This design has the advantage of bypass problems with population stratification. Indeed, the main disadvantage of case-control studies, the most common approach in genetic studies, is unaccounted population admixture with a possible threat to the validity of the obtained results.

## Methods

This study included samples from four Brazilian states: Minas Gerais state (Center for Rehabilitation of Craniofacial Anomalies, Dental School, University of José Rosário Vellano, Alfenas-MG), located in the Southeastern region of Brazil, Paraná state (Association of Carrier of Lip and Palate Cleft-APOFILAB, Cascavel-PR), in the Southern region, Paraíba state (University Hospital of Lauro Wanderley-HULW, João Pessoa-PB) and Bahia state (Santo Antonio Hospital, Salvador-BA), which are located in the Northeast region. The cohort was composed of 223 trios composed of one affected offspring and two healthy parents. Information on demographic characteristics, including gender and race, and family history of oral cleft and cancer were collected through direct interview of the patients and/or mothers (Additional file 1: Table S1). All patients were diagnosed independently and screened for the presence of associated anomalies or syndromes by the team of specialists from each center, and only patients with the nonsyndromic form were included. The NSCL ± P was classified with the incisive foramen as reference [41].

The genomic DNA was isolated from buccal mucosa cells obtained by mouthwash with a 3% sucrose solution, using a salting-out protocol previously described [42]. SNPs, including rs16260, rs9929218, rs7186053 and rs4783573 in *CDH1* and rs7591, rs7210356, rs4791171, rs11079571 and rs3923087 in *AXIN2* (Table 1), were genotyped using the Taqman genotyping assay (assay-on-demand, Applied Biosystems). Genotyping analyses were randomly repeated in 10% of the samples, and the concordance rate was 100%. These SNPs were selected based on their minor allele frequencies (www.ncbi.nlm.nih.gov) and because they were described as risk factors for oral cleft and breast and gastric cancer [29, 37, 39, 40, 43–46]. The study was approved by the ethics review board of each of the centers or hospitals affiliated with the collaborative study. Written informed consent was obtained from the parents or guardians and/or the participants.

**Table 1:** Characteristics of the single-nucleotide polymorphisms (SNPs)

SNP	Gene	Chromosome	Location	Position	Alleles
rs16260	*CDH1*	16	promoter	68,737,131	C/**A**
rs9929218	*CDH1*	16	intron	68,787,043	G/**A**
rs7186053	*CDH1*	16	intron	68,805,390	G/**A**
rs4783573	*CDH1*	16	intron	68,806,685	G/**A**
rs7591	*AXIN2*	17	3’ UTR	65,528,964	A/**T**
rs7210356	*AXIN2*	17	intron	65,532,832	G/**A**
rs4791171	*AXIN2*	17	intron	65,545,379	A/**G**
rs11079571	*AXIN2*	17	intron	65,552,563	G/**A**
rs3923087	*AXIN2*	17	intron	65,553,143	A/**G**

Risk alleles in bold

For allele transmission analysis, the transmission disequilibrium test (TDT) was performed with the aid of the FBAT (Family Based Association Test) software. The haplotype-based analysis was conducted using the HBAT (Haplotype-Based Association Test) command in FBAT software. These tests are intended to verify the genotype-phenotype association from the unbalanced transmission of the alleles and haplotypes from healthy parents to affected children. The Z-score value measures the deviation from the null hypothesis, and positive Z-score indicates that the allele is over-transmitted to the affected offspring (is a risk factor), whereas a negative Z-score indicates under-transmission to affected offspring (is a protective factor). The pair-wise linkage disequilibrium (LD) was estimated from the combined data of all trios calculating D’ and r^2^ using the using Haploview software (v4.2). The *p* value of <0.05 was considered statistically significant.

## Results

Our results showed that the minor A allele of *AXIN2* rs7210356 was significantly under-transmitted to patients with NSCL ± P (*p* = 0.01) (Table 2). HBAT analyses showed the C-G-A-A and A-G-A-G haplotypes formed by rs16260, rs9929218, rs7186053 and rs4783573 in *CDH1* were significantly transmitted from parents to the children with NSCL ± P (*p* = 0.01 and *p* = 0.008, respectively) (Table 3). In *AXIN2* gene, the frequency of the T-G-G-A-G haplotype formed by rs7591, rs7210356, rs4791171, rs11079571 and rs3923087 SNPs was also under-transmitted to patients with NSCL ± P (*p* = 0.03) (Table 4).

**Table 2:** Transmission disequilibrium test (TDT) of the single-nucleotide polymorphisms (SNPs) in nonsyndromic cleft lip with or without cleft palate (NSCL ± P) trios

SNP	MAF	Number of families	T/NT	Z score	*p* value
rs16260	0.295	135	86/49	1.00	0.31
rs9929218	0.271	133	78/55	-0.46	0.64
rs7186053	0.329	134	84/50	-0.98	0.32
rs4783573	0.523	146	103/43	-1.39	0.16
rs7591	0.543	154	121/33	0.55	0.58
rs7210356	0.081	54	34/20	-2.47	**0.01**
rs4791171	0.616	139	118/21	0.36	0.71
rs11079571	0.215	99	50/49	-1.68	0.09
rs3923087	0.606	136	116/20	0.00	1.00

*MAF:* minor allele frequency, *T/NT:* transmission/non-transmission counts

**Table 3:** Haplotype family-based association test for *CDH1* rs16260, rs9929218, rs7186053 and rs4783573 performed using HBAT

Haplotype^*^	Number of families	Frequency	Z score	*p* value
C-G-G-G	119	0.336	0.73	0.46
C-G-G-A	94	0.216	0.80	0.42
A-A-A-A	82	0.185	-0.71	0.47
C-G-A-A	42	0.064	-2.48	**0.01**
A-G-A-G	22	0.032	2.66	**0.008**
C-A-G-G	17	0.031	-0.07	0.94
C-G-A-G	21	0.029	-1.20	0.23
A-A-G-A	20	0.025	0.64	0.52
C-A-G-A	16	0.018	-0.96	0.34
A-A-G-G	12	0.015	-0.14	0.88
A-G-G-G	10	0.013	-0.31	0.76
A-A-A-G	12	0.010	1.12	0.26

^*^Sequence: rs16260, rs9929218, rs7186053 and rs4783573

**Table 4:** Haplotype family-based association test for *AXIN2* rs7591, rs7210356, rs4791171, rs11079571 and rs3923087 performed using HBAT

Haplotype^*^	Number of families	Frequency	Z score	*p* value
T-G-G-G-G	96	0.486	1.45	0.15
A-G-A-G-A	77	0.158	0.75	0.45
A-G-A-A-A	62	0.121	0.02	0.98
A-G-G-G-G	30	0.052	0.35	0.73
A-A-A-A-A	17	0.031	-1.89	0.06
T-G-G-G-G	20	0.030	0.15	0.88
A-A-A-G-G	15	0.029	-0.37	0.71
A-G-A-G-G	14	0.019	-1.39	0.16
T-G-G-A-G	12	0.016	-2.08	**0.03**
A-G-G-A-G	11	0.013	0.39	0.69

^*^Sequence: rs7591, rs7210356, rs4791171, rs11079571 and rs3923087

The LD pattern of the SNPs examined is displayed in Table 5. The LD values between *CDH1* SNPs were low, whereas *AXIN2* SNPs rs7591, rs7210256 and rs4791171 were in complete linkage disequilibrium (rs7591 and rs7210356: *D*’ = 0.97 and *r* ^2^ = 0.10, rs7591 and rs4791171: *D*’ = 0.93 and *r* ^2^ = 0.68, rs7210356 and rs4791171: *D*’ = 0.95 and *r* ^2^ = 0.12).

**Table 5:** Linkage disequilibrium analysis of the *CDH1* (A) and *AXIN2* (B) SNP. D’ coefficient is above the diagonal and r’ is below diagonal

A					
	rs16260	rs9929218	rs7186053	rs4783573	
rs16260	-	0.77	0.69	0.49	
rs9929218	0.55	-	0.57	0.59	
rs7186053	0.41	0.26	-	0.48	
rs4783573	0.09	0.12	0.10	-	
B					
	rs7591	rs7210356	rs4791171	rs11079571	rs3923087
rs7591	-	0.97	0.93	0.78	0.77
rs7210356	0.10	-	0.95	0.35	0.27
rs4791171	0.68	0.12	-	0.74	0.79
rs11079571	0.20	0.04	0.23	-	0.86
rs3923087	0.47	0.01	0.61	0.31	-

## Discussion

Although the exact environmental and genetic risk factors associated with NSCL ± P remains unclear, the understanding of the genetic mechanisms involved in this malformation are evolving. Interestingly, recent studies have demonstrated a relationship between congenital malformations and malignancies, and genetic alterations in *AXIN2* and *CDH1* are associated with both NSCL ± P [37, 39, 47] and cancer of breast and gastric [21, 28, 29, 31–34]. In this study we explored the clinical relevance of breast and gastric cancer-associated polymorphisms in *AXIN2* and *CDH1* with NSCL ± P risk in the Brazilian population. Our results suggest that the *AXIN2* rs7210356 SNP is associated with the occurrence of NSCL ± P in the investigated population. This SNP was associated with breast cancer risk in premenopausal but not in postmenopausal women [29]. The impact of the rs7210356 polymorphism in lip and palate development is unclear and our findings are of interest especially to understand this association. Haplotype analysis showed that five SNPs in *AXIN2* (rs7591, rs7210356, rs4791171, rs11079571 and rs3923087) were associated with occurrence of NSCL ± P. Previously studies revealed that the both rs7591 and rs3923087 were associated with NSCL ± P and tooth agenesis [37, 46].

*CDH1* haplotypes composed by rs16260, rs9929218, rs7186053 and rs4783573 were associated with NSCL ± P in our study population. Many reports showed that rs16260 is associated with the development of gastric cancer [45, 48], but it is controversial its association with craniofacial development [39, 40]. Similarly, rs9929218 was shown to have a borderline association with unilateral NSCL/P in Brazilian population [37] as well as the haplotype composed of rs9929218, rs7186053, rs4783573 and rs16958383 to NSCL/P in the population of Warsaw [40]. *CDH1* plays a key role in cell adhesion, which is vital to normal development, including craniofacial morphogenesis and palatal fusion [49]. Thus, further examination of haplotypes in *CDH1* gene, including rs16260, rs9929218, rs7186053 and rs4783573, is needed to identify the molecular relevant causes.

## Conclusions

In summary, little is known about the relationship between *AXIN2* and *CDH1* genes and oral clefts in humans. In the present study identified one SNP and one haplotype in *AXIN2* gene and two haplotypes in *CDH1* gene significantly associated with NSCL ± P susceptibility in Brazilian population. Except the C-G-A-A haplotype formed by rs16260, rs9929218, rs7186053 and rs4783573 in *CDH1* gene which was associated with increased risk of NSCL ± P, the others haplotypes in *AXIN2* and *CDH1* genes, as well as the rs7210356 allele alone, were associated in a protective manner to NSCL ± P. Though, the population size in our study is small and the significant results need to be confirmed in larger groups with known family history of breast and gastric cancer to better understand the interactions between *AXIN2* and *CDH1* genes in the development of NSCL ± P.

## Additional file

Additional file 1: Table S1. Clinical and epidemiological characteristics of patients with nonsyndromic cleft lip with or without cleft palate. (DOCX 14 kb)

## Abbreviations

AXIN2: Axis inhibition protein 2
CDH1: Cadherin-1
FBAT: Family based association test
HBAT: Haplotype-based association test
MAF: Minor allele frequency
NSCL ± P: Nonsyndromic cleft lip with or without cleft palate
NSCLO: Nonsyndromic cleft lip only
NSCLP: Nonsyndromic cleft lip and palate
NSCPO: Nonsyndromic cleft palate only
OR: Odds ratio
SNP: Single nucleotide polymorphism
T/NT: Transmission/non-transmission counts
TDT: Transmission disequilibrium test
