# Supplementary material for: Clinical relevance of breast and gastric cancer-associated polymorphisms as potential susceptibility markers for oral clefts in the Brazilian population
Source: BMC Med Genet. 2017 Apr 4;18:39. doi: 10.1186/s12881-017-0390-y (PMC5379638; doi:10.1186/s12881-017-0390-y)
Supplement: Additional file 1: Table S1. — Clinical and epidemiological characteristics of patients with nonsyndromic cleft lip with or without cleft palate. (DOCX 14 kb) [file 12881_2017_390_MOESM1_ESM.docx]

**Additional file 1: Table S1** Clinical and epidemiological characteristics of patients with

nonsyndromic cleft lip with or without cleft palate.

|  | Cleft lip  n (%) | Cleft lip and palate  n (%) | *p* value |
| --- | --- | --- | --- |
| Gender |  |  |  |
| Male | 36 (56.2) | 99 (62.3) |  |
| Female | 28 (43.8) | 60 (37.7) | 0.40 |
| Race/ethnicity |  |  |  |
| Caucasian | 36 (56.3) | 100 (62.9) |  |
| Non-Caucasian | 28 (43.7) | 59 (37.1) | 0.35 |
| Orofacial cleft in 1^st^ degree relative |  |  |  |
| Yes | 22 (34.4) | 55 (34.6) |  |
| No | 42 (65.6) | 104 (65.4) | 0.97 |
| Cancer in 1^st^ degree relative |  |  |  |
| Yes | 33 (51.6) | 69 (43.4) |  |
| No | 31 (48.4) | 90 (56.6) | 0.27 |
| Numbers of cancer occurences |  |  |  |
| One type of cancer | 20 (60.6) | 57 (82.6) |  |
| Two type of cancer | 12 (36.4) | 10 (14.5) |  |
| Three type of cancer | 1 (3.0) | 2 (2.9) | 0.04 |
| Type of cancer |  |  |  |
| Breast | 9 (19.1) | 9 (10.9) |  |
| Gastric | 6 (12.8) | 6 (7.2) |  |
| Others* | 32 (68.1) | 68 (81.9) | 0.19 |

***Cancer in bladder, bone, bowel, colon, esophagus, head and neck, kidney, liver, lung, oral cavity, oropharynx, pancreas, prostate, skin and uterus, and leukemia.
